# Overall Anxiety Severity and Impairment Scale (OASIS) and Overall Depression Severity and Impairment Scale (ODSIS): Adaptation and Validation in Buenos Aires, Argentina

**DOI:** 10.32872/cpe.10451

**Published:** 2023-06-29

**Authors:** Rodrigo Lautaro Rojas, Camila Florencia Cremades, Milagros Celleri, Cristian Javier Garay

**Affiliations:** 1 Faculty of Psychology, Universidad de Buenos Aires, Buenos Aires, Argentina; Philipps-University of Marburg, Marburg, Germany

**Keywords:** anxiety, depression, adaptation, validation, psychometrics

## Abstract

**Background:**

The OASIS and ODSIS scales are two transdiagnostic brief 5-item instruments designed to assess the severity and functional impairment associated with symptoms of anxiety and depression, respectively. The present study aimed to adapt and validate the online versions of both scales in Buenos Aires, Argentina.

**Method:**

A sample of 344 women and men from the general population of Buenos Aires completed a test battery consisting of the OASIS, the ODSIS, the Beck Depression Inventory (BDI), the Beck Anxiety Inventory (BAI), the Positive and Negative Affect Scale (PANAS) and the Multicultural Quality of Life Index (MQLI). Descriptive statistics and item discrimination of both scales were analyzed, as well as their factorial structure, internal consistency, and convergent and discriminant validity, using the R programming language.

**Results:**

The results showed a unidimensional factorial structure, excellent internal consistency, and adequate construct validity for both the OASIS and the ODSIS.

**Conclusion:**

These results supports the use of both scales as valid and reliable instruments to assess severity and interference due to anxiety and depression in the general population of Buenos Aires, Argentina.

## Highlights

- Both scales are valid and reliable instruments for the assessment and detection of anxiety and depressive symptoms.
- Their availability is important for the reliable application of the Unified Protocol in our country.
- They can be used in our context in an online format without compromising their psychometric properties.

Emotional disorders (Barlow, 1991) are the most frequent psychological problems in the Argentinian population. The lifetime prevalence of anxiety disorders reaches 16.4% and for major depressive disorder it reaches 8.7%, while their annual prevalence reaches 9.4% and 3.8%, respectively (Stagnaro et al., 2018). Additionally, both groups of disorders are costly (Parés-Badell et al., 2014; Ruiz-Rodríguez et al., 2017), interfering (Kazdin & Blase, 2011; Olatunji et al., 2007) and highly comorbid problems (Brown et al., 2001; Brown & Barlow, 2009).

There are multiple tools to assess general anxiety and depression, such as the Beck Anxiety Inventory (BAI; Beck et al., 1988; Argentinian adaptation by Vizioli & Pagano, 2020) or the Beck Depression Inventory (BDI; Beck et al., 1996; Argentinian adaptation by Brenlla & Rodríguez, 2006). Similarly, there are also numerous instruments to assess symptoms associated with specific anxiety disorders, such as the Penn State Worry Questionnaire (PSWQ; Meyer et al., 1990; Argentinian adaptation by Rodríguez Biglieri & Vetere, 2011) for generalized anxiety disorder, and the Panic Disorder Severity Scale (PDSS; Shear et al., 1997) for panic disorder, not yet adapted to our setting.

However, all of these instruments are limited to assessing the frequency and intensity of specific symptoms and do not offer a global measure of the severity and interference associated with these symptoms, either in established disorders or at subclinical levels (González Robles et al., 2018; Norman et al., 2006). Scales of this type do not adequately reflect the impact of symptoms on functioning (Bentley et al., 2014) and are of little use in assessing the overall impact of treatment (Ito, Oe, et al., 2015).

Similarly, while scales designed to assess specific symptoms of specific diagnoses are ideal for detailed assessments, they are less useful in clinical settings when assessing comorbid cases (Campbell-Sills et al., 2009). Additionally, the use of different scales can be time-consuming and impractical in settings such as primary care (Campbell-Sills et al., 2009; Osma et al., 2019).

In view of these problems, two scales have been developed to capture the severity and interference of anxious and depressive symptomatology in a brief and transdiagnostic manner–that is, regardless of the diagnostic category of these symptoms: the Overall Anxiety Severity and Impairment Scale (OASIS; Norman et al., 2006) and the Overall Depression Severity and Impairment Scale (ODSIS; Bentley et al., 2014).

The OASIS is a brief scale designed to assess the severity and interference associated with anxiety. It can be used with individuals with one or more anxiety disorders or with anxiety symptoms below the diagnostic threshold. It consists of 5 items referring to the past week and it’s scored on a Likert-type scale ranging from 0 to 4. Higher scores indicate greater anxiety-related severity and impairment. Severity is captured by items that ask for the frequency and intensity of anxiety symptoms (e.g., "2. In the last week, when you have felt anxious, how intense or severe was your anxiety?"), while interference is measured by items that assess the impact of these symptoms on work/school and social life. It also includes an item that evaluates avoidance as a specific symptom of anxiety. In its original version, it yielded a mean of 7.16 (*SD* = 3.05), excellent internal consistency (α = .80), a unifactorial structure and excellent convergent validity in a non-clinical sample (Norman et al., 2006).

The scale was developed to capture common domains of all anxiety disorders in a fast and simple way in demanding clinical settings such as primary care (González-Robles et al., 2018), and to monitor changes in symptoms over the course of treatment (Campbell-Sills et al., 2009). It was validated in both clinical and non-clinical samples and in paper-and-pencil and online formats, showing excellent internal consistency and good convergent and discriminative validity (Bragdon et al., 2016; Campbell-Sills et al., 2009; Farrahi et al., 2020; González-Robles et al., 2018; Hermans et al., 2015; Ito, Oe, et al., 2015; Moore et al., 2015; Norman et al., 2006; Norman et al., 2011; Osma et al., 2019; Osma et al., 2021; Sandora et al., 2021). Different cut-off scores have been proposed to discriminate between people with clinical and subclinical anxiety in their different validations (see Table 1).

**Table 1:** Validations of the OASIS

Authors	Country	Sample	Format	*M* (*SD*)	Cutoff points
Bragdon et al. (2016)	USA	Clinical sample (*N* = 202)	Paper-and-pencil	AD: 9.63 (*SD* = 4.69) WAD: 4.96 (*SD* = 4.26)	–
Campbell-Sills et al. (2009)	USA	Clinical sample (*N* = 1036)	Paper-and-pencil	10.77 (*SD* = 4.02)	8
Farrahi et al. (2020)	Iran	Students sample (*N* = 464)	Paper-and-pencil	4.83 (*SD* = 3.68)	–
González-Robles et al. (2018)	Spain	Clinical sample (*N* = 583)	Online	8.69 (*SD* = 4.21)	7.5
Hermans et al. (2015)	Netherlands	Clinical sample (*N* = 257)	Paper-and-pencil	AD: 8.46 (*SD* = 3.96) WAD: 3.00 (*SD* = 3.51)	5
Ito, Oe, et al. (2015)	Japan	Clinical (*N* = 1667) and Non-clinical sample (*N* = 1163)	Online	Clinical: 9.69 (*SD* = 5.55) Non-clinical: 5.56 (*SD* = 4.91)	9
Moore et al. (2015)	USA	Clinical sample (*N* = 347)	Paper-and-pencil	9.35 (*SD* = 4.38)	8
Norman et al. (2006)	USA	Students sample (*N* = 711)	Paper-and-pencil	7.16 (*SD* = 3.05)	–
Norman et al. (2011)	USA	Students sample (*N* = 171)	Paper-and-pencil	6.61 (*SD* = 4.01)	8
Osma et al. (2019)	Spain	Clinical sample (*N* = 339)	Paper-and-pencil	10.45 (*SD* = 4.49	10
Osma et al. (2021)	Spain	Students sample (*N* = 382)	Online	3.92 (*SD* = 4.13)	4
Sandora et al. (2021)	Czech Republic	Non clinical sample (*N* = 2912)	Online	9.50 (*SD* = 4.25)	15

*Note*. AD = Anxiety disorders; WAD = Without anxiety disorders; *SD* = Standard deviation.

The ODSIS was developed based on the OASIS in order to capture the severity and interference associated with depressive symptoms. It maintains the same structure of 5 items, which refer to the last week and are scored on a Likert-type scale ranging from 0 to 4, with higher scores indicating greater severity and functional interference associated with depression (Bentley et al., 2014). Like the OASIS, its items assess the frequency and intensity of depressive symptoms and their interference with work/school and social life (e.g., "5. In the past week, how much has depression interfered with your social life and relationships?"). The most notable difference is that the OASIS item assessing avoidance was replaced by one assessing interference due to loss of interest and difficulty experiencing pleasure as a symptom of depression. In its original version, it yielded a mean of 5.50 (*SD* = 5.04), excellent internal consistency (α = .94), a unifactorial structure, and adequate convergent and discriminant validity in the clinical subsample (Bentley et al., 2014).

This scale was designed to be used across mood disorders and with depressive symptoms below the diagnostic threshold (Bentley et al., 2014). It was validated in clinical and non-clinical samples and in paper-and-pencil and online formats, showing excellent internal consistency and good convergent and discriminative validity (Bentley et al., 2014; Ito, Bentley, et al., 2015; Mira et al., 2019; Osma et al., 2019; Osma et al., 2021; Sandora et al., 2021). Different cut-off scores have been proposed to discriminate between people with clinical and subclinical depression in their different validations (see Table 2).

**Table 2:** Validations of the ODSIS

Authors	Country	Sample	Format	*M* (*SD*)	Cutoff points
Bentley et al. (2014)	USA	Clinical sample (*N* = 100) Students sample (*N* = 566) Community sample (*N* = 189)	Paper-and-pencil	5.50 (*SD* = 5.04) 2.57 (*SD* = 3.36) 5.16 (*SD* = 4.81)	8
Ito, Bentley, et al. (2015)	Japan	Clinical (*N* = 1667) and Non-clinical sample (*N* = 1163)	Online	Clinical: 8.68 (*SD* = 6.32) Non-clinical: 3.67 (*SD* = 4.87)	5
Mira et al. (2019)	Spain	Clinical sample (*N* = 474)	Online	7.83 (*SD* = 4.90)	5
Osma et al. (2019)	Spain	Clinical sample (*N* = 339)	Paper-and-pencil	9.87 (*SD* = 5.14)	10
Osma et al. (2021)	Spain	Students sample (*N* = 382)	Online	2.79 (*SD* = 4.06)	5
Sandora et al. (2021)	Czech Republic	Non-clinical sample (*N* = 2912)	Online	8.73 (*SD* = 4.34)	12

*Note*. *M* = Mean; *SD* = Standard deviation.

The administration of instruments in online format has increased in recent years, due to advantages such as accessibility and ease of administration and scoring (van Ballegooijen et al., 2016). Although paper and online versions of the same instrument often correlate strongly, mean scores and psychometrics may differ (Alfonsson et al., 2014), so specific validations need to be conducted for online administration. Both the OASIS and ODSIS were developed in paper-and-pencil format, and their online use requires specific validation in this format, as was conducted in other media (González-Robles et al., 2018; Mira et al., 2019).

Considering that both anxiety disorders and depression are highly prevalent, comorbid and often associated with significant distress and interference, it is necessary to have transdiagnostic measures to capture the severity and interference associated with anxious and depressive symptomatology in our local environment. Although there are instruments designed to assess symptoms of anxiety and depression that have been adapted and validated in our setting, none of them can quickly capture the severity and social and occupational interference associated with such symptomatology. The present study aims to carry out the linguistic, cultural and psychometric adaptation of the online versions of the OASIS and ODSIS scales in the population of Buenos Aires, Argentina.

## Method

### Linguistic and Cultural Adaptation

The adaptation of both instruments was carried out taking into consideration the recommendations of the International Test Commission (ICT) for the adaptation of tests to other cultures (Muñiz et al., 2013). The translation into Spanish was carried out following a direct translation method by five independent translators and five judges who evaluated the quality of the translations on a Likert scale from 1 (quite different) to 4 (identical). The translations that received the highest number of high scores (3 or 4) on the Likert scale from the judges were selected to form the preliminary versions of both scales.

With the preliminary version of the instrument, a pilot test was carried out with a sample of 12 individuals using Google Forms, in which the comprehension of the items was evaluated and a first analysis of the items was carried out. Participants signed an informed consent form expressing their voluntary participation. The final adapted versions of both instruments can be found in Appendices A and B (see Supplementary Materials).

### Procedure

The psychometric properties of the translated and culturally adapted versions of the OASIS and the ODSIS were analysed. The recruitment of participants was non-probabilistic using the snowball method through the dissemination of flyers on social media. All participants gave their consent to participate in the study in which the confidentiality of the data, the purposes of the research and the possibility of withdrawing from the study at any time were clarified. All participants then completed a set of scales through a virtual Google Forms questionnaire.

### Participants

The sample consisted of 344 adults (18-65 years old) from the general population residing in the City of Buenos Aires (26.7%, *N* = 92), Greater Buenos Aires (49.1%, *N* = 169) and the Province of Buenos Aires (24.1%, *N* = 83), Argentina. The mean age of the sample was 29.44 (*SD* = 10.62). The 80.5% identified with the female gender (*N* = 277), 19.2% with the male gender (*N* = 66) and the remaining 0.3% with a fluid gender (*N* = 1). In terms of educational level, 56.1% had completed secondary school (*N* = 193), 43.3% had completed university (*N* = 149) and 0.6% had completed primary school (*N* = 2).

### Instruments

#### Socio-Demographic Questionnaire

As part of the test battery, an ad-hoc questionnaire was included in which the participants' age, gender, place of residence and level of education were asked.

#### Beck Depression Inventory II (BDI II)

The BDI-II (Beck et al., 1996; Argentinian adaptation by Brenlla & Rodríguez, 2006) is an inventory designed to assess depressive symptoms. It consists of 21 items referring to the past week and is scored on a Likert-type scale from 0 (not at all) to 3 (severely). The higher the score, the greater the severity of the depressive symptomatology. The validation in our setting showed an adequate internal consistency with a Cronbach's alpha coefficient of .88.

#### Beck Anxiety Inventory (BAI)

The BAI (Beck et al., 1988; Argentinian adaptation by Vizioli & Pagano, 2020) is composed of 21 items that assess the severity of anxiety symptoms. Each item refers to specific anxiety symptoms and is scored on a Likert-type scale from 0 (not at all) to 4 (it bothered me a lot). Higher scores indicate greater severity of the anxiety symptomatology. Its validation in the local setting yielded a Cronbach's alpha coefficient of 0.93.

#### Brief Positive and Negative Affect Schedule (PANAS)

The PANAS (Thompson, 2007; Argentinian adaptation by Moriondo et al., 2012) is an instrument designed to dimensionally measure positive and negative affect. In the present study, the short version of the instrument designed by Thompson (2007) and adapted to Argentina by Moriondo et al. (2012) was selected, consisting of four subscales: trait positive affect (five items), trait negative affect (five items), state positive affect (five items) and state negative affect (five items). Each item is scored on a Likert-type scale from 1 (very little or not at all) to 5 (very much or completely). It was adapted in our context with a Cronbach's alpha coefficient of .73 (.84 for negative affect and .75 for positive affect).

#### Multicultural Quality of Life Index (MQLI)

The MQLI (Mezzich et al., 1996; Argentinian adaptation by Jatuff et al., 2007) is a self-administered instrument designed to assess quality of life in a brief, multicultural and multidimensional way. It consists of 10 items assessing different aspects of quality of life, each of which is scored on a Likert-type scale from 1 (poor) to 10 (excellent). All sub-dimensions are summed to produce the Global Quality of Life Index. The higher the score, the higher the quality of life perceived. It was adapted to our setting with a Cronbach's alpha of .85.

#### Overall Anxiety Severity and Impairment Scale (OASIS)

The OASIS (Norman et al., 2006) is a brief scale designed to measure the severity and interference associated with anxiety symptoms. It consists of 5 items inquiring about the frequency and intensity of anxiety symptoms, the interference caused by anxiety symptoms in school/work and social life and avoidance as a specific symptom of anxiety. Each item consists of 5 response options on a Likert-type scale from 0 (little or none) to 4 (extreme). It was adapted to Spanish in Spain with a Cronbach's alpha of .86 (González-Robles et al., 2018).

#### Overall Depression Severity and Impairment Scale (ODSIS)

The ODSIS (Bentley et al., 2014) is a brief scale designed to measure the severity and interference associated with depressive symptoms. It consists of 5 items inquiring about the frequency and intensity of depressive symptoms, the interference caused by depressive symptoms in school/work and social life and the difficulty experiencing pleasure and/or interest as a specific symptom of depression. Each item consists of 5 response options on a Likert-type scale ranging from 0 (little or none) to 4 (extreme). It was adapted to Spanish in Spain with a Cronbach's alpha of .92 (Mira et al., 2019).

## Data Analysis

All analyses were carried out using the R programming language. First, the sociodemographic characteristics of the sample (*N* = 344) and the descriptive statistics (mean, variance, skewness and kurtosis) of both OASIS and ODSIS items were analysed.

Prior to the analysis of the internal structure of both scales, the existence of adequate intercorrelation between items was assessed using the Kaiser-Meyer-Olkin measure of sampling adequacy and Bartlett's test of sphericity. To analyse the factor structure, a Confirmatory Factor Analysis was carried out. Following Norman et al. (2006) and Bentley et al. (2014), a one-factor model was tested for both scales. The fit of the models was assessed using the Comparative Fit Index (CFI), the Tucker-Lewis Index (TLI) and the Standardised Mean Squared Error (SRMR) as criteria. The following cut-off scores were used to determine a good fit: CFI and TLI around .90 and SRMR below 0.08 (Marsh et al., 2004).

For the analysis of internal consistency, both Cronbach's Alpha and Omega Coefficients were calculated (Dunn et al., 2014). Convergent and discriminant validity was explored by calculating Pearson's *r* correlations between the OASIS and ODSIS and well-established measures of anxiety (BAI), depression (BDI), positive and negative affect (PANAS) and quality of life (MQLI). To interpret the correlation values, the *p*-value was calculated and the benchmarks for *r*-values proposed by Hinkle et al. (2003) were used. *r*-values between .90 and 1.00 were considered very high, those between .70 and .90 were considered high, those between .50 and .70 were considered moderate and those between .30 and .50 were considered low. Corrected item-total correlations were also calculated to analyze the discrimination of the items of both scales.

We also wanted to explore the existence of differences in the scores of both scales regarding gender. For this purpose, a Student's *t*-test for independent samples was performed. Because the criteria of normality and homoscedasticity of variances were not met in all groups, a Wilcoxon test was also performed. Finally, a linear regression was performed to determine whether age was a good predictor of change in severity levels of depression and anxiety.

## Results

### Descriptive Analysis of the Items

The mean score of the OASIS in the sample analysed was 6.52 (*SD* = 3.90). The mean, variance, skewness and kurtosis of each item were analysed. All items had skewness and kurtosis values between -1 and 1, suggesting a normal distribution (see Table 3).

**Table 3:** Mean, Standard Deviation, Skewness, and Kurtosis of OASIS Items

Item	*M*	*SD*	Skewness	Kurtosis
*1*	1.88	0.96	0.38	-0.43
*2*	1.62	0.86	0.01	-0.32
*3*	0.96	1.03	1	0.54
*4*	1.98	0.95	0.75	0.06
*5*	0.99	1.04	0.83	-0.06

As for the ODSIS, the mean score in the sample analysed was 4.48 (*SD* = 4.40). All items had skewness and kurtosis values between -1 and 1.03, suggesting a normal distribution (see Table 4).

**Table 4:** Mean, Standard Deviation, Skewness, and Kurtosis of ODSIS Items

Item	*M*	*SD*	Skewness	Kurtosis
*1*	0.96	0.98	0.92	0.45
*2*	0.88	0.92	0.72	-0.19
*3*	0.89	1.05	1.02	0.2
*4*	0.77	0.93	1.03	0.15
*5*	0.77	1	1.02	1

### Item Discrimination Analysis

Item discrimination was calculated using corrected item-total correlations. All OASIS items showed to discriminate adequately (*r* > .30) [Item 1 (*r* = .66), Item 2 (*r* = .68), Item 3 (*r* = .65), Item 4 (*r* = .73), Item 5 (*r* = .67)]. Similarly, the ODSIS items also showed adequate discrimination (*r* > .30) [(Item 1 (*r* = .84), Item 2 (*r* = .83), Item 3 (*r* = .87), Item 4 (*r* = .84), Item 5 (*r* = .81)].

### Internal Structure Analysis

First, the existence of adequate intercorrelation between items was assessed using the Kaiser-Meyer-Olkin measure of sampling adequacy and Bartlett's test of sphericity, obtaining evidence suggesting the feasibility of conducting a factor analysis for both the OASIS (*KMO* = .83; χ^2^ = 227.86, *gl* = 10, *p* < .001) and the ODSIS (*KMO* = .87; χ^2^ = 452.48, *gl* = 10, *p* < .001). Confirmatory factor analysis (CFA) was then conducted on the one-factor model proposed in previous research for the OASIS (Norman et al., 2006) and ODSIS (Bentley et al., 2014). Model fit was determined by the Comparative Fit Index (CFI = .991 for the OASIS; CFI = .999 for the ODSIS), the Tucker-Lewis Index (TLI = .982 for the OASIS; TLI = .997 for the ODSIS) and the standardised root mean square error (SRMR = .061 for the OASIS; SRMR = .031 for the ODSIS), obtaining adequate goodness-of-fit indices.

### Internal Consistency Analysis

For the analysis of internal consistency, Cronbach's alpha coefficient was calculated, obtaining a value of α = .90 for the OASIS and α = .97 for the ODSIS. The Omega coefficient yielded a value of ω = .93 for the anxiety scale and ω = .93 for the depression scale.

### Convergent and Discriminant Validity

Pearson's *r* correlations between the OASIS, the ODSIS and related scales are shown in Table 5. A high and significant positive association was found between the OASIS and the ODSIS, *r*(343) = .70, *p* < .01, the BDI, *r*(343) = .70, *p* < .01, and between the OASIS and the BAI, *r*(343) = .73, *p* < . 01. A moderate and significant positive association was found between the OASIS and the negative trait affectivity, *r*(343) = .61, *p* < .05, and state, *r*(343) = .54, *p* < .05, subscales of the PANAS. On the other hand, a moderate and significant negative association was found between the OASIS and the MQLI, *r*(343) = -.66, *p* < .01, and a low and significant negative association between the OASIS and the positive trait affectivity, *r*(343) = -.46, *p* < .05, and state, *r*(343) = -.42, *p* < .01, subscales of the PANAS.

**Table 5:** Correlations Between OASIS and ODSIS and Other Scales

	OASIS	ODSIS	BDI	BAI	MQLI	PANAST NA	PANAST PA	PANASS NA	PANASS PA
OASIS	–	.70**	.70**	.73**	-.66*	.61*	-.46*	.54*	-.42**
ODSIS	.70**	–	.73**	.62**	-.65**	.51**	-.49**	.46**	-.40**

*Note*. OASIS = Overall Anxiety Severity and Impairment Scale; ODSIS = Overall Depression Severity and Impairment Scale; BDI = Beck Depression Inventory; BAI = Beck Anxiety Inventory; MQLI = Multicultural Quality of Life Index; PANAST = Positive and Negative Affect Scale Trait; PANASS = Positive and Negative Affect Scale State; NA = Negative Affect; PA = Positive Affect.

*p < .05. **p < .01.

A high and significant positive association was found between ODSIS and BDI, *r*(343) = .73, *p* < .01, a moderate and significant positive association between ODSIS and BAI, *r*(343) = .62, *p* < .01, and the negative trait affectivity subscale, *r*(343) = .51, *p* < .01, of the PANAS and a low and significant positive association with the negative state affectivity subscale, *r*(343) = .46, *p* < .01. On the other hand, a moderate and significant negative association was found between the ODSIS and the MQLI, *r*(343) = -.65, *p* < .01, and a low and significant negative association between the ODSIS and the positive trait, *r*(343) = -.49, *p* < .01, and state, *r*(343) = -.40, *p* < .01, subscales of the PANAS.

### Differences According to Gender and Age

Differences in OASIS and ODSIS scores were assessed regarding gender. A *t*-test was conducted to compare the OASIS and ODSIS scores of those who reported identifying with the female gender and those who reported identifying with the male gender to explore the existence of significant gender differences. It was found that females scored significantly higher than males on both the OASIS, *t*(107) = -2.76, *p* < .01, and ODSIS, *t*(117) = -2.91, *p* < .01. Considering that the assumption of normality in the groups was not met, a Wilcoxon test was also performed, which also yielded statistically significant differences for OASIS, *W* = 10935, *p* < .05, and ODSIS, *W* = 10783; *p* < .05.

Finally, to assess whether age functioned as a good predictor of anxiety severity and interference, a linear regression was performed taking the OASIS score as the dependent variable and age as the predictor variable. It was found that the higher the age, the lower the severity and interference due to anxiety, β = -0.10, *F*(1, 342) = 27.75, *p* < .001, *R*^2^ = .07. The same procedure was performed to determine whether age functioned as a good predictor of severity and interference due to depression, finding that the older the age the lower the severity and interference due to depression, β = -0.10, *F*(1, 342) = 23.13, *p* < .001, *R*^2^ = .06.

## Discussion

The aim of the present study was to carry out the adaptation and validation of the OASIS and ODSIS in the Argentine population in an online format. The psychometric validation included the analysis of item discrimination, factorial structure, internal consistency, convergent and discriminant validity, and differences in scores as a function of sociodemographic variables for both scales.

Considering only those adaptations that took participants from the general population, both the OASIS (*M* = 6.52; *SD* = 3.90) and the ODSIS (*M* = 4.48; *SD* = 4.40) yielded mean scores higher than those obtained in the Japanese (Ito, Oe, et al., 2015; Ito, Bentley, et al., 2015) adaptations, but lower than those obtained in the Czech study (Sandora et al., 2021). The latter may be due to the fact that in the Czech study the data were collected during the COVID-19 pandemic, which may have influenced the scores obtained. Also, the ODSIS yielded higher mean scores than those obtained in the non-clinical subsample of the original validation (Bentley et al., 2014). The higher scores obtained in local adaptations compared to Japanese or American ones may be linked to the high prevalence of problems linked to anxiety and depression in Argentina (Stagnaro et al., 2018).

On the other hand, taking into account the adaptations that were performed in online format, as expected the local adaptations presented lower scores than those that took a clinical sample (González-Robles et al., 2018; Mira et al., 2019) but higher than the one that took a sample of students (Osma et al., 2021). However, all the above comparisons should be taken with caution because there have been no studies investigating the cross-cultural measurement invariance of these scales.

The 5 items of both scales were found to discriminate adequately (*r* > .30), indicating that they allow to distinguish between people with different levels of severity and interference due to anxiety and depression, respectively.

As in previous research (Bentley et al., 2014; Norman et al., 2006; Osma et al., 2019), confirmatory factor analysis revealed a unidimensional factor structure with strong factor loadings for all items of both scales. Regarding reliability, both the OASIS and the ODSIS demonstrated excellent internal consistency in the sample of Argentinian participants (α = .90 and ω = .93. for the OASIS and α = .97 and ω = .93. for the ODSIS), showing values similar to those of previous validations performed in the general population (Bentley et al., 2014; Ito, Bentley, et al., 2015; Ito, Oe, et al., 2015; Sandora et al., 2021).

Regarding construct validity, significant positive correlations were found between the OASIS and the BAI and between the ODSIS and the BDI, providing evidence for the convergent validity of both scales with two of the most widely used instruments for the assessment of anxiety and depression. The fact that significant positive correlations were also found between the OASIS and the ODSIS, the BDI and the PANAS subscales of trait and state negative affect, but lower than that found for the BAI, is interpreted as evidence of the discriminant validity of the instrument. Likewise, the fact that significant positive correlations were also found between the ODSIS and the OASIS, the BAI and the negative trait and state affect subscales of the PANAS, but lower than that found in relation to the BDI, is interpreted as evidence of the instrument's discriminant validity. Taken together, these findings provide evidence of adequate construct validity for both the OASIS and the ODSIS, in agreement with previous research (González-Robles et al., 2018; Mira et al., 2019; Osma et al., 2019; Osma et al., 2021).

In contrast to previous adaptations (González-Robles et al., 2018; Ito, Bentley, et al., 2015; Ito, Oe, et al., 2015; Mira et al., 2019), significant differences were found in the OASIS and ODSIS total scores as a function of gender and age. Individuals who identified with the female gender scored significantly higher on both scales than males, which is consistent with previous literature that indicates that Argentinian women are 85% more likely to suffer from anxiety disorders than men (Stagnaro et al., 2018). Furthermore, in line with the research by Stagnaro et al. (2018), which reported a higher prevalence of emotional disorders in younger individuals, it was found that the levels of severity and interference due to anxiety and depression decrease with increasing age. The older the age, the lower the severity and interference due to anxiety and depression.

In sum, the results of the present study are consistent with those obtained in previous validations performed in the general population (Bentley et al., 2014; Ito, Bentley, et al., 2015; Ito, Oe, et al., 2015; Sandora et al., 2021), and support the OASIS and ODSIS scales as valid and reliable instruments to assess the severity and functional interference due to anxiety and depression in the general population of Buenos Aires, Argentina.

This is the first study to evaluate the psychometric properties of the OASIS and ODSIS scales in Argentina. Having instruments adapted to our environment that allow us to measure the severity of anxiety and depression and their level of interference in daily functioning is essential to assess and detect both groups of disorders, which are highly prevalent in our population (Stagnaro et al., 2018), whether they occur in isolation or in comorbidity, both in clinical and non-clinical settings. Their availability is also a first step for the reliable application of the Unified Protocol, a transdiagnostic treatment designed to address emotional disorders that uses both scales to measure the patient's change in anxiety and depressive symptomatology on a weekly basis (Barlow et al., 2011).

Furthermore, and in line with previous research (González-Robles et al., 2018; Ito, Bentley, et al., 2015; Ito, Oe, et al., 2015; Mira et al., 2019), the results also suggest that both the OASIS and the ODSIS can be used in our setting in an online format without compromising their psychometric properties. Having adapted instruments in online format is important because it enables their use in the context of internet-based interventions, which have proliferated in recent decades in the field of cognitive-behavioral therapies (Andersson et al., 2019). The development of these interventions is especially important in Argentina, where access to evidence-based treatments is difficult and the inclusion of the technology in academia is still scarce (Distéfano et al., 2015). The availability of both scales in online format represents a contribution to this promising field in Argentina.

### Limitations

Limitations of the study include the fact that the sample consisted of people from the general population of Buenos Aires, which limits the generalizability of the results to clinical settings and people from another regions of the country. In addition, no methods were used to guarantee whether the participants were receiving psychological treatment or have an actual mental disorder. Also, the mean age of the participants was very young and the educational level very high, which may have been related to the method chosen to reach them.

Another limitation was that the proportion of males and females was not balanced, which may have affected the representativeness of the results. Unlike previous studies (Sandora et al., 2021), the comparison between men and women was performed without having calculated measurement invariance between both genders because the sample size was smaller than recommended in the literature (<100) to calculate it (Meade & Bauer, 2007; Putnick & Bornstein, 2016). Finally, unlike previous adaptations, test-retest reliability, sensitivity to change and cut-off scores for both scales could not be established in our population. It would be desirable for future research to consider these aspects and analyse them in a clinical sample.

## Supplementary Materials

The Supplementary Materials contain the following items (for access see Index of Supplementary Materials below):

- *Appendix A:* presents the Argentine version of the Overall Anxiety Severity and Impairment Scale (OASIS)
- *Appendix B:* presents the Argentine version of the Overall Depression Severity and Impairment Scale (ODSIS).

RojasR. L.
CremadesC. F.
CelleriM.
GarayC. J.
 (2023). Supplementary materials to "Overall Anxiety Severity and Impairment Scale (OASIS) and Overall Depression Severity and Impairment Scale (ODSIS): Adaptation and validation in Buenos Aires, Argentina"
[Argentine versions of the OASIS and ODSIS]. PsychOpen. 10.23668/psycharchives.12903
PMC1050825337732148

## Data Availability

Materials and analysis code for this study are available by emailing the corresponding author.
